# A One-Dimensional Dynamic Constitutive Modeling of Ethylene Vinyl Acetate (EVA) Foam

**DOI:** 10.3390/polym15234514

**Published:** 2023-11-24

**Authors:** Jin Wu, Fude Lu, Jiaxuan Chen, Mingqi Wang

**Affiliations:** School of Packaging and Material Engineering, Hunan University of Technology, Zhuzhou 412007, China; wujin2023555@163.com (J.W.); 13103920669@163.com (J.C.); a18822783829@163.com (M.W.)

**Keywords:** ethylene-vinyl acetate, foams, strain rate effect, entrapped air, constitutive model

## Abstract

Ethylene vinyl acetate copolymer (EVA) is good for impact protection and energy absorption, and belongs to rate sensitive-dependent materials. This study aimed to investigate the influence of increased strain rate and the presence of entrapped air on the enhancement of foam material strength. The compression deformation behavior of EVA foams containing a microporous structure was extensively investigated over different strain rates of 0.0017/s, 0.033/s, and 0.17/s, where each test was conducted at a constant compression velocity. A one-dimensional dynamic constitutive model was established to describe the large deformation response of EVA to different strain rates. The model included two components, the material action part and the air pressure part. Quasi-static and dynamic compression tests were used to determine the constitutive relations of three parameters, $a_{1}$, $a_{2}$, and the leaking rate $\overset{\cdot }{\delta }$. The samples with EVA foams at different strain rates were fitted using ORIGIN software, and the constitutive model parameters were obtained. It was found that the ratio of the air leaking rate to the strain rate gradually decreases, causing air within the EVA to be trapped in the cells rather than escaping in a timely manner with increasing strain rates.

## 1. Introduction

Polymeric foams are extensively employed for their energy-absorbing properties [1,2,3]. Among these, ethylene vinyl acetate copolymer (EVA) foam stands out as an environmentally friendly and closed-cell polymeric foam with a microporous structure [4]. EVA foam exhibits notable attributes such as being lightweight, having great surface resilience, low energy absorption, high energy return, and remarkable cushioning and shock absorption capabilities, as well as notable thermal insulation and resilience. It has widespread applications in cushioning materials, notably in areas such as shoe insoles [5], soft foam pads, and foam packaging [6]. With the increasing demand for environmentally friendly and lightweight materials, research on the mechanical properties of energy-absorbing materials has received increasing attention in recent years. Through the examination of mechanical properties, scientists strive to comprehend the deformation behavior, strength, stiffness, and energy absorption capacity exhibited by these materials. This knowledge helps design the sizes and structures of materials that can effectively mitigate impact forces, reduce vibrations, and enhance overall safety.

When employed in packaging, foams play a crucial role in safeguarding the packaged items by effectively absorbing vibrations and attenuating shocks, ensuring their safe transportation without sustaining any damage. Given the high deformation rates involved in this application, it becomes imperative to possess a comprehensive understanding of the compressive constitutive laws governing foams. Such knowledge is indispensable for the design of packaging systems, enabling accurate prediction of the dynamic behavior exhibited by foams. During dynamic compressive loading, the resistance force exerted by foams comprises two main components: (1) deformation of the elastomeric foam structure, which exhibits nonlinear viscoelastic behavior, and (2) the generation of air pressure [2]. In open-cell foams, this pressure arises from the flow of air through the porous foam structure, while in closed-cell foams, it results from the accumulation of gas pressure within the individual cells. Gibson and Ashby [7] investigated the deformation behavior of closed-cell polyethylene foams under dynamic compression, and found that closed-cell foam materials generally exhibit strain rate sensitivity. They analyzed the reasons for the increase in platform stress. Shen et al. [8] argues that when the strain rate increases, foam cells lack sufficient time to undergo complete deformation during static compression. Additionally, the collapse modes of foam under static and dynamic compression differ, and they proposed a mechanism to explain foam cell collapse. Dannemann and Lankford [9] studied the effect of strain rate on the strength of polymeric foams, and their findings show that the strength increases with higher strain rates. They concluded that the viscous effect caused by air flow through the pore network plays a significant role. Srivastava et al. [10] examined the main factors affecting the performance of closed-cell foams, including air pressure, substrate properties, cell geometry, and foam fabrication methods. In contrast, they found that air pressure had no significant impact on open-cell foams. Additionally, Sadot et al. [11] found that the effect of the air inside the cell in aluminum foam on the dynamic response of aluminum foam is dependent on the strain rate.

In order to predict the kinetic response of cushioning materials, phenomenological constitutive models of specific foam materials based on quasi-static and dynamic com-pression experiments are effective methods [12,13,14]. Zhang et al. [15] established phenomenological ontological equations that incorporate the effects of strain rate and temperature on the constitutive behavior of polymeric foam materials. Yang et al. [16] developed a constitutive equation capable of describing the visco-hyperelastic behavior of compressible elastomeric materials under high strain rates. Anani and Alizadeh [17] developed a constitutive equation that incorporates the strain rate-dependency of elastomeric materials, enabling accurate prediction of the structural response of these materials under dynamic loadings, even at large strains exceeding 100%. Jeong [18] proposed a constitutive equation for incorporating the strain rate effect in elastomeric materials utilizing a shape function multiplied by a four-parameter modulus function, which demonstrated improved approximation of experimental data compared to the previous model employing a two-parameter modulus function. Su et al. [19] developed a pressure-dependent phenomenological constitutive model, a stress potential function, for transversely isotropic foams, incorporating the characteristic stress and strain. Li et al. [20] developed a phenomenological, large-deformation, isotropic viscoelastic constitutive model for closed-cell EVA foam, specifically focusing on the rate-dependent, large deformation mechanical behavior. Wen et al. [21] developed a dynamic constitutive model for high-density RPUF, suitable for large deformation, high pressure, and high strain rates. However, the studies fail to adequately account for the influence of the air trapped within the foam, despite its significant role in foam behavior. Xu et al. [22] investigated the air leaking rate within the pores of aluminum foam and established a corresponding instantaneous equation, considering the influence of strain rate. The investigation of air within the cells of polymer foam has been limited, primarily attributed to the inherent confinement of air within the intricate and interconnected pore structure of the foam, leading to challenges in characterizing and quantifying this air. The complex nature of the foam’s small-scale pore network presents difficulties in the precise characterization and measurement of the enclosed gases, contributing to the scarcity of studies in this area.

A comprehensive understanding of the deformation mechanism exhibited by foam structures during the loading process is of utmost importance, as the mechanical properties of foams are heavily influenced by their deformation behavior. Notably, one significant factor impacting these mechanical properties is the presence of intra-pore air within the foam structure. However, the dense internal structure inherent to polymer foams presents inherent challenges when attempting to design experiments aimed at investigating the direct influence of internal air on the foam’s mechanical properties. Consequently, this study made enhancements to the traditional phenomenological mechanical model by introducing parameters for air leakage rate and strain rate. The primary objective is to characterize the mechanical behavior of ethylene vinyl acetate (EVA) foam influenced by air within the cells. Specifically, this model considers the substantial influence of intra-pore air. Through the examination of the air leaking rate $\overset{\cdot }{\delta }$, this investigation aims to explore the consequential effect caused by the air trapped within the foam’s pores on its structural integrity.

## 2. Materials and Methods

### 2.1. Materials and Specimens

The foam materials used in this research are commercial Jiexin low-density EVA foam boards. The tested samples of EVA foams were made using a cutting tool. In order to investigate the compressive strain rate dependency of EVA foam (average density of 35 kg/m^3^), samples with the dimensions of 70 mm × 70 mm × 30 mm (length × width × thickness) were prepared for quasi-static load compression testing and dynamic load compression testing.

### 2.2. Equipment and Test Setup

The experimental setup employed for the tests was a Zwick/Roell Z250 materials testing machine (made by Zwick in Ulm, Germany). The Zwick/Roell Z250 machine has a load capacity of 250 kN, and can reach a velocity of up to 600 mm/min for compression tests. The loading method employed in this study involved continuous loading using a fixed indenter. The specimens were placed on the lower fixed platen. During the compression, the upper platen moved downwards to crush the specimens. Compression tests were performed on specimens at three different loading velocities: 3 mm/min, 60 mm/min, and 300 mm/min, using the loading head. The maximum compression volume was set at approximately 24 mm for the test. Each set of tests was conducted in five replicates under identical conditions, maintaining room temperature throughout. The average stress–strain curve was derived from the force–displacement data obtained in all of the experiments, taking into account the dimensions of the specimens tested.

### 2.3. Data Processing

According to the above experimental test, the position value and load value of the sample during the test can be obtained. The strain of the foam during compression, ε, can be calculated with the following:

(1) $$\varepsilon =\frac{L(t)-L_{0}}{L_{0}},$$

where $L_{0}$ and L(t) are the initial thickness of the foam and the position value when compression time reaches a certain value t, respectively. Since L(t) is a function of the compression time, in differentiating Equation (1) with respect to t we obtain the following:

(2) $$\overset{\cdot }{\varepsilon }=\frac{d\varepsilon }{dt}=\frac{1}{L_{0}}\cdot \frac{dL(t)}{dt}=\frac{v(t)}{L_{0}},$$

where $\overset{\cdot }{\varepsilon }$ is the strain rate and v(t) is loading velocity. Therefore, these loading rates of 3 mm/min, 60 mm/min, and 300 mm/min resulted in strain rates of 0.0017/s, 0.033/s, and 0.17/s, respectively.

The stress of the foam during compression, σ, can be calculated with the following:

(3) $$\sigma =\frac{W}{A},$$

where W is the compression load and A is the cross-sectional area of the sample.

Applying the energy efficiency method [23], the plateau stress, $\sigma^{*}{}_{\mathrm{pl}}$, can then be calculated by

(4) $$\sigma^{*}{}_{\mathrm{pl}}=\frac{\int_{0}^{\varepsilon_{d}}\sigma (\varepsilon )d\varepsilon }{\varepsilon_{d}},$$

where $\varepsilon_{d}$ represents the densification strain of the foam during its compression.

## 3. Results and Discussion

### 3.1. Quasi-Static and Dynamic Compression

**Figure 1:**
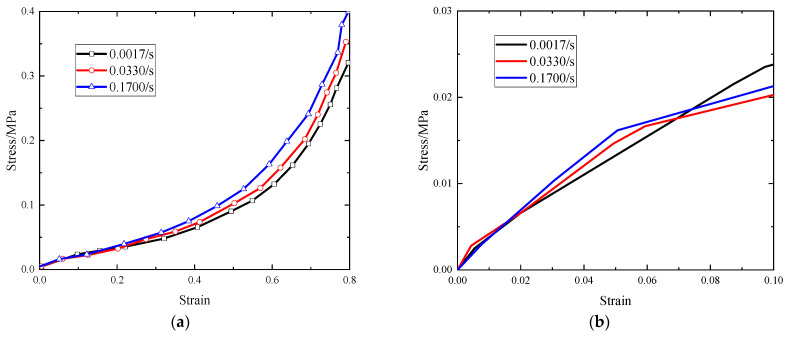
Stress–strain responses of EVA foam with different compressive strain rates: (**a**) complete stress–strain responses; (**b**) stress–strain curve in the linear elastic regime.

In Figure 1, stress–strain curves under different strain rate conditions are shown. According to Figure 1a, although tests of higher strain rates are not available because of test condition restrictions, EVA foam has obvious significant strain rate sensitivity. The stress–strain curve obtained from the compression testing of the foam exhibits three distinct stages, namely the linear elastic stage, the plateau stage, and the densification stage. Furthermore, a clear enhancement in the stress–strain response can be observed as the compressive strain rate is increased. With an increase in the strain rate, there is a corresponding increase in the stress for a given strain, resulting in higher energy absorption by the EVA foam. According to Figure 1b, there is negligible discrepancy in the stress–strain curves of EVA foam at different strain rates during the elastic deformation stage. This implies that the strain rate has minimal influence on EVA foam’s behavior during this stage. This can be attributed to the fact that the primary deformation occurring during the elastic deformation stage takes place within the EVA foam substrate, while the cell structure of EVA foam remains largely undeformed, resulting in limited impact from the air confined within the pores. However, upon entering the stress plateau stage, the EVA foam cell structure starts to deform, leading to a more pronounced interplay between the foam substrate and the air within the pores. Consequently, it can be concluded that the strain rate sensitivity of EVA foam, as well as other closed-cell polymer foams, stems fundamentally from the interaction between the pore air and the foam substrate during the stress plateau stage.

### 3.2. EVA Foam Energy Absorption Diagram

The energy absorption diagram is a significant tool utilized in engineering and materials science to comprehend and assess the energy-absorbing characteristics of various materials. This diagram provides a comprehensive representation of a material’s response to external forces, and how it dissipates energy during deformation processes. By examining the energy absorption diagram, researchers and engineers can gain valuable insights into the material’s performance under different loading conditions, including impact, compression, and dynamic loading scenarios. In essence, the energy absorption diagram graphically illustrates the relationship between applied mechanical energy and the corresponding deformation response of the material. It allows researchers to identify the critical points where the material exhibits specific behaviors, such as yielding, strain hardening, and failure, which are vital factors that influence energy absorption capabilities. In practical applications, energy absorption diagrams play a crucial role in material selection and design optimization. By analyzing these diagrams, engineers can identify materials that possess superior energy absorption properties suitable for specific engineering challenges, such as crashworthiness in automotive components or impact protection in protective gear. Furthermore, the energy absorption diagram aids in understanding the mechanisms of energy dissipation within the material during deformation, thereby guiding the development of novel materials with enhanced energy-absorbing characteristics. This scientific and logical approach enables researchers to make informed decisions and develop innovative materials for a wide range of applications, ensuring safer and more efficient engineering solutions in various industries.

The energy absorption capacity of a material, W, can be quantified by calculating the energy absorption per unit volume, as defined in Equation (5).

For this study, a sample of EVA with a density of 35 kg/m^3^ was chosen, and it was subjected to various loading rates, specifically 0.0017/s, 0.033/s, and 0.017/s. These loading rates were used to investigate the material’s response to different strain rates and understand its energy-absorbing behavior under dynamic loading conditions.

(5) $$W=\int_{0}^{\varepsilon }\sigma \left(\varepsilon \right)d\varepsilon$$

**Figure 2:**
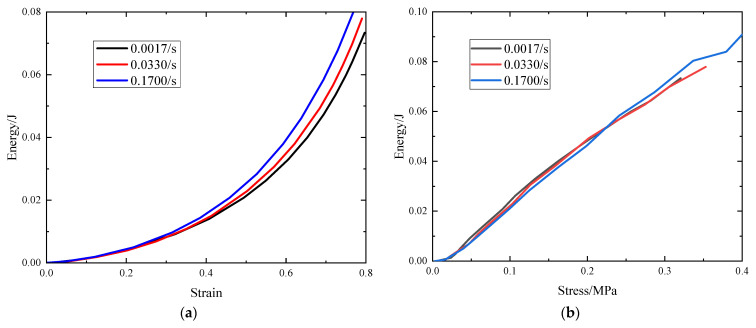
Energy absorption diagram: (**a**) energy absorption–strain curve; (**b**) energy absorption–stress curve.

Figure 2a presents the energy absorption plot, depicting the relationship between the absorbed energy (W) and stress for the EVA foams tested at different strain rates. The curves in the plot display variations in energy absorption with respect to stress levels for distinct strain rates. Figure 2 reveals that as the strain rate increases, the foam absorbs more energy for the same strain, indicating a positive correlation between the strain rate and energy absorption. This trend indicates that at higher strain rates, the EVA foam can absorb more energy during dynamic loading, which is reflected in its improved energy-absorbing properties.

The energy absorption efficiency is another important parameter used to characterize a material’s ability to absorb energy. It is defined as the ratio of absorbed energy to stress, $E_{\mathrm{ff}}$, as shown in Equation (6). This parameter provides valuable insights into the material’s energy dissipation capabilities under varying stress levels, allowing for a comprehensive assessment of its energy absorption performance.

(6) $$E_{\mathrm{ff}}=\frac{\int_{0}^{\varepsilon }\sigma \left(\varepsilon \right)d\varepsilon }{\sigma }$$

**Figure 3:**
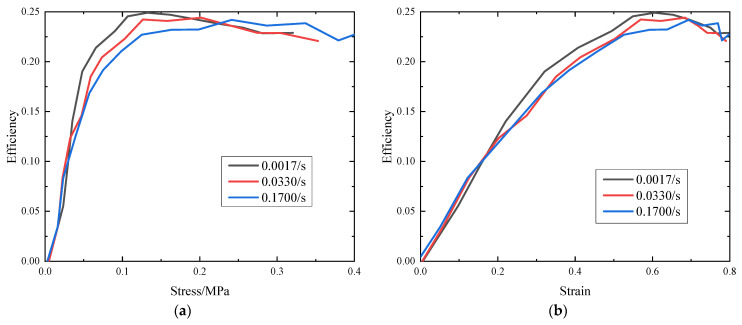
Energy absorption efficiency diagram: (**a**) energy absorption efficiency–stress curve; (**b**) energy absorption efficiency–strain curve.

Plotting the energy absorption efficiency of EVA as a function of the stress at different strain rates yields a series of curves, as shown in Figure 3a. The efficiency always has a maximum at a certain stress, because beyond a certain stress level, the increase in absorbed energy is lower than the corresponding stress increase [24]. Indeed, as evidenced from Figure 3, the energy absorption efficiency of the EVA foam declines with higher strain rates. Additionally, in Figure 3b, it becomes apparent that the energy absorption efficiency of EVA foams at various strain rates begins to decline as the strain approaches approximately 0.6. This behavior is attributed to the foam compression entering the densification stage, leading to changes in its energy absorption characteristics. During the initial stages of compression, namely the linear and plateau phases, the energy absorption efficiency shows an upward trajectory, implying that the foam is relatively more effective in absorbing energy at lower strain rates. However, as the compression progresses into the densification stage, the energy absorption efficiency starts to decrease. This occurrence is attributed to the unique behavior of the foam during densification, wherein significant deformation and rearrangement of the internal structure lead to a decrease in its energy absorption efficiency. Furthermore, Figure 3 illustrates that at higher strain rates, there is a decrease in the energy absorption efficiency at the same strain or stress level, suggesting that the strain rate indeed influences the energy absorption efficiency.

### 3.3. Establishment of One-Dimensional Dynamic Constitutive Modeling

#### 3.3.1. Phenomenological Mechanical Model

The rheological model of foam is a mathematical representation used to describe the viscoelastic behavior of foam materials under deformation. It aims to capture the mechanical response of foams, taking into account their unique microstructure and complex interactions between the gas phase and the solid phase. The rheological model typically involves constitutive equations that relate stress, strain, and strain rate to characterize the foam’s viscoelastic properties. One commonly used rheological model for foams is the Maxwell model [17], which combines a spring and dashpot in series to represent the foam’s elastic and viscous responses, respectively. Another widely applied model is the Kelvin–Voigt model, which consists of a spring and dashpot in parallel. These models provide insights into the foam’s response to dynamic loading and relaxation processes. Such phenomenological mechanical models assume the existence of an “equivalent material”.

**Figure 4:**
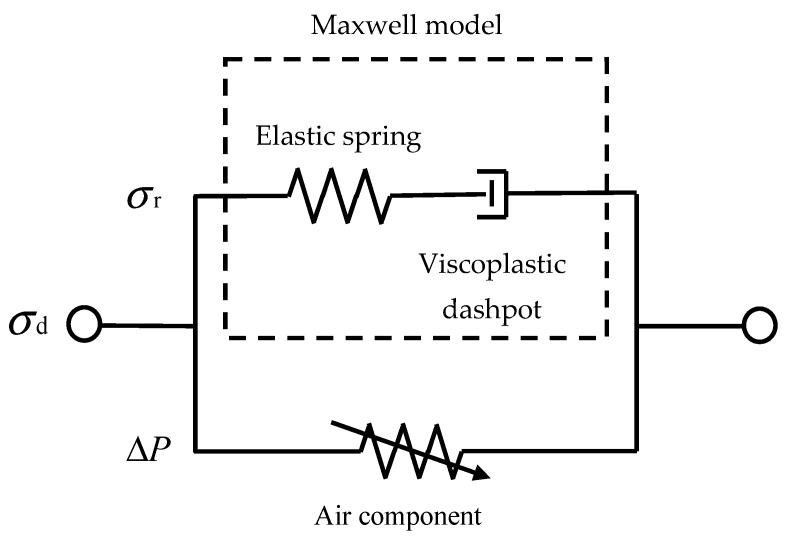
Phenomenological mechanical model.

The experimental results lead to the conclusion of the viscoelastic–plastic mechanical behavior of EVA. The relationship between viscoelastic–plastic EVA microphase separation structure and mechanical deformation is complex. The behavior of EVA under compressive loading can be described using rheological models for two specific materials, elastoplastic solids and air components (simulated viscous), as shown in Figure 4. One of them, the elastoplastic solid, can be simulated with the Maxwell model. The Maxwell model combines a spring (representing the elastic response) and a dashpot (representing the viscous or plastic response) in series. When the foam undergoes deformation, the spring component stores elastic energy, while the dashpot component dissipates energy through viscous or plastic deformation. The Maxwell model is often combined with the Kelvin model to develop more comprehensive rheological models that can describe the viscoelastic plasticity of foams more accurately. However, to better capture the air action in foam, air components are incorporated into the rheological model. These air components can represent the viscous action of foam, taking into account the effects of air flow and pressure within the foam’s porous structure.

**Figure 5:**
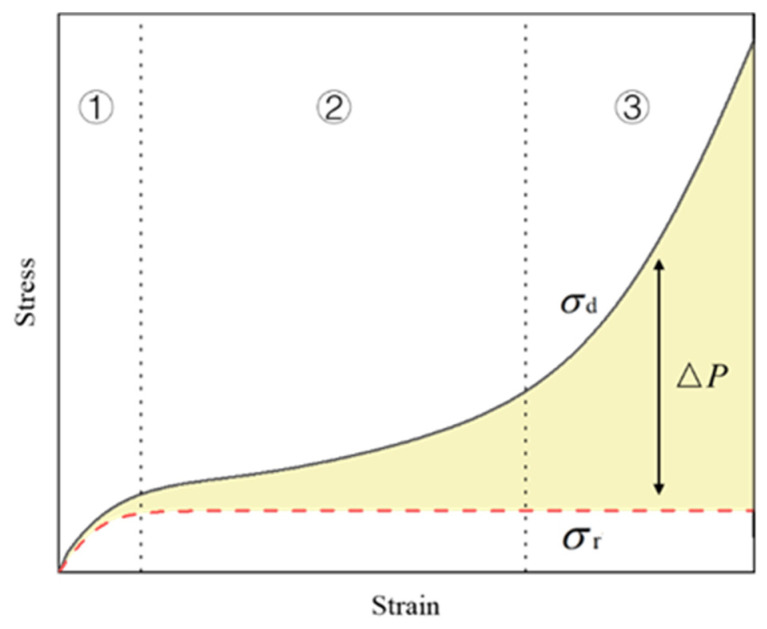
Stress-strain response of EVA foam as affected by material and in-cell air.

In Figure 5, the stress–strain curve of EVA foam is shown. The stress–strain curve of EVA foam has three stages, namely the linear elastic stage, the plateau stage, and the densification stage. During the compression process, the primary factors of interest are the foam substrate and the entrapped air confined within the pores. To account for the influence of the air present in the pores of EVA foam on its material properties, the dynamic stress $\sigma_{d}$ can be mathematically described with the following:

(7) $$\sigma_{d}=\sigma_{r}+\Delta P,$$

where $\sigma_{r}$ ($\sigma_{r}=\sigma^{*}{}_{\mathrm{pl}}$) is the dynamic plateau stress and ΔP is the pressure variation attributed to the air confined within the cells during the process of foam compression. Figure 5 illustrates the schematic diagram depicting the relationship and interactions among variables $\sigma_{d}$, $\sigma_{r}$, and ΔP.

#### 3.3.2. One-Dimensional Dynamic Constitutive Modeling of EVA

Lu et al. [25] proposed a simplified elastic–plastic model for foams, which describes the stress–strain relationship in the uniaxial case as follows:

(8) $$\sigma =a_{1}\cdot \tanh(a_{2}\cdot \varepsilon )+\frac{a_{3}\cdot \varepsilon }{a_{4}-\varepsilon },$$

where $a_{1}=\sigma_{Y}$, and $\sigma_{Y}$ represent yield stress of the foam at reference strain rate. In addition, $a_{2}=\frac{E}{\sigma_{Y}}$, and E represent elastic modulus of the foam at reference strain rate. The slope is equal to be parameter E near the origin of the stress–strain responses. As the strain increases, the stress-strain curve gradually approaches a horizontal asymptote, indicating the attainment of a stable state at larger strains. Following Lu’s argument [26], the first term accurately fits the linear elastic stage and the plateau stage. Therefore, the dynamic plateau stress, $\sigma_{r}$, can be calculated as follows:

(9) $$\sigma_{r}=a_{1}\cdot \tanh(a_{2}\cdot \varepsilon ).$$

During the compression process, the presence of air within the cells of the EVA foam gives rise to the build-up of internal pressure. However, a portion of the air escapes and leaks out. In accordance with the observations put forth by Zhang and Yu [27], it is postulated that neglecting volume changes arising from buckling and assuming an isothermal compression process allows for the quantification of air leaking transpiring within the cells as a consequence of foam deformation. The leaking of the air during the compression process, δ, can be defined by the following:

(10) $$\delta =1-\frac{PV}{P_{0}V_{0}},$$

where $P_{0}$ and P are the initial pressure (atmospheric pressure) and the pressure when the strain reaches a certain value, ε, respectively; $V_{0}$ and V are the initial volume and the volume when the strain reaches ε, respectively. The pressure P can be calculated with the following:

(11) $$P=P_{0}\cdot \frac{1-\delta }{1-\varepsilon },$$

since δ is a function of crushing time, *t*, differentiating Equation (11) with respect to *t* and taking $\overset{\cdot }{\varepsilon }=\frac{v}{L_{0}}=\frac{\partial \varepsilon }{\partial t}$, can obtain,

(12) $$\overset{\cdot }{P}=\frac{P_{0}}{1-\varepsilon }\cdot \left(\frac{1-\delta }{1-\varepsilon }\cdot \overset{\cdot }{\varepsilon }-\overset{\cdot }{\delta }\right).$$

It’s evident that the rate of leakage and air pressure change is influenced by both the strain rate and the strain.

Since $\overset{\cdot }{P}=\frac{\partial P}{\partial t}=\frac{\partial P}{\partial \varepsilon }\cdot \frac{\partial \varepsilon }{\partial t}=\frac{\partial P}{\partial \varepsilon }\cdot \overset{\cdot }{\varepsilon }$, Equation (12) can be re-written into another form, which is

(13) $$\frac{\partial P}{\partial t}-\frac{1}{1-\varepsilon }P=\frac{P_{0}\overset{\cdot }{\delta }}{\overset{\cdot }{\varepsilon }}\cdot \frac{1}{1-\varepsilon }.$$

Since the strain rate is constant, Equation (9) is a first-order linear differential equation, which has a solution,

(14) $$P=\frac{1}{1-\varepsilon }\cdot \left(1-\frac{\overset{\cdot }{\delta }}{\overset{\cdot }{\varepsilon }}\cdot \varepsilon \right).$$

The parameter, ΔP, exhibits a correlation with both the strain rate and air leaking rate, $\overset{\cdot }{\delta }$, during the compression process, and its macroscopic relationship can be mathematically described by

(15) $$\Delta P=P_{0}\cdot \left(\frac{1}{1-\varepsilon }-1\right)\cdot \left(1-\frac{\overset{\cdot }{\delta }}{\overset{\cdot }{\varepsilon }}\right).$$

Therefore, the one-dimensional dynamic instantaneous equation of the EVA foam, taking into account the influence of the air trapped within the cells, can be calculated by

(16) $$\sigma_{d}=a_{1}\cdot \tanh(a_{2}\cdot \varepsilon )+P_{0}\cdot \left(\frac{1}{1-\varepsilon }-1\right)\cdot \left(1-\frac{\overset{\cdot }{\delta }}{\overset{\cdot }{\varepsilon }}\right).$$

From Equation (16), The derived one-dimensional dynamic principal structure model for EVA foam consists of three parameters that require identification, namely $a_{1}$, $a_{2}$ and $\overset{\cdot }{\delta }$.

### 3.4. Application and Results of One-Dimensional Dynamic Constitutive Modeling

Since the first term of Equation (16) accurately fits the linear elastic stage and the plateau stage, parameters $a_{1}$ and $a_{2}$ were fitted using the compression test data with a minimum strain rate of 0.0017/s (set as the reference strain rate). Then, the fitting of parameter $\overset{\cdot }{\delta }$ was performed using test data acquired under various strain rates.

**Figure 6:**
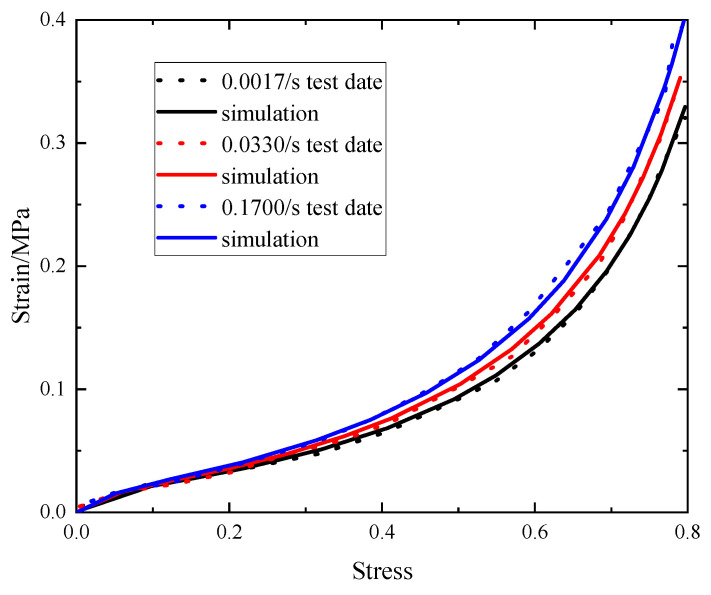
Stress–strain responses of EVA foam.

The samples with a density of 35 kg/m^3^ at strain rates of 0.0017/s, 0.033/s, and 0.17/s were fitted using ORIGIN software (2023), and the constitutive model parameters and their R^2^ of fitting were obtained, which are listed in Table 1. Figure 6 demonstrates a favorable agreement between the fitting points and the experimental test results.

The compressive stress–strain data of EVA serve as a fundamental basis for its utilization in the design of energy-absorbing cushioning materials and the simulation analysis of its application in specialized fields such as collisions and impacts. By considering the intrinsic characteristics of EVA and utilizing stress–strain curves derived from compression tests, an effective constitutive model is established, and the model parameters are determined. This enables designers to select appropriate stress–strain curves that correspond to the desired compression strain rate when encountering different impact conditions. By inputting the corresponding strain rate into the constitutive model, designers can readily obtain the corresponding stress–strain curve without the need for additional compression tests.

### 3.5. Analysis of One-Dimensional Dynamic Model Parameters Results

**Figure 7:**
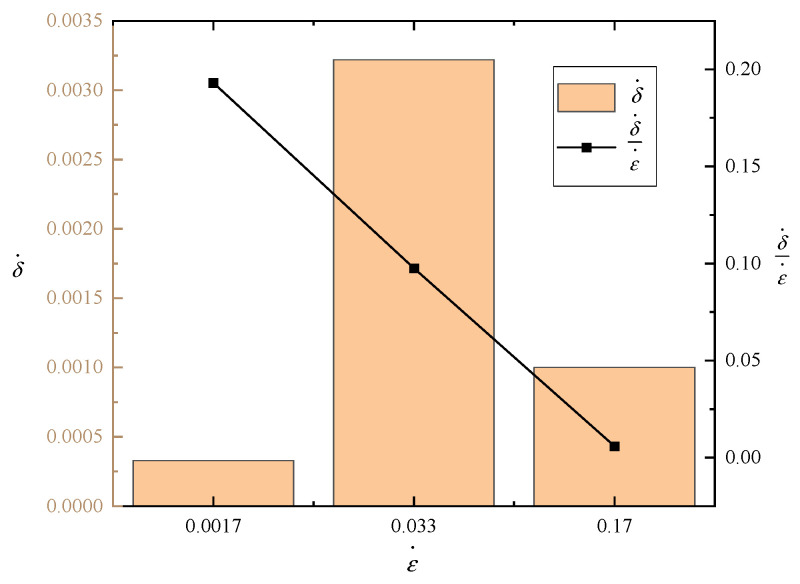
Parameters $\overset{\cdot }{\delta }$ and $\frac{\overset{\cdot }{\delta }}{\overset{\cdot }{\varepsilon }}$ at different strain rates.

Based on the observations from Figure 7, it is evident that the parameter $\overset{\cdot }{\delta }$ representing the presence of air within the EVA cells varies with different strain rates. This finding aligns with the conclusion that the value of $\overset{\cdot }{\delta }$ is influenced by the parameter $\overset{\cdot }{\varepsilon }$. During the compression tests conducted at three different strain rates, the behavior of $\overset{\cdot }{\delta }$ does not exhibit a discernible regularity. The relationship between $\overset{\cdot }{\delta }$ and $\overset{\cdot }{\varepsilon }$ is not simply characterized by a positive or negative correlation. However, it is observed that the ratio of $\overset{\cdot }{\delta }$ to $\overset{\cdot }{\varepsilon }$, $\frac{\overset{\cdot }{\delta }}{\overset{\cdot }{\varepsilon }}$, decreases as the value of $\overset{\cdot }{\varepsilon }$ increases. If the air within the cells were to spill out uniformly, it would be expected that $\overset{\cdot }{\delta }$ would show a linear increase with $\overset{\cdot }{\varepsilon }$, resulting in a constant ratio between the two. However, it is evident that the observed ratio, $\frac{\overset{\cdot }{\delta }}{\overset{\cdot }{\varepsilon }}$, deviates from this expected pattern, indicating that the spilling of air within the cells is not uniform. This observation suggests that as $\overset{\cdot }{\varepsilon }$ increases, the corresponding $\overset{\cdot }{\delta }$ for the air within the cells does not adjust at the same rate. Therefore, a part of the air fails to escape promptly and remains trapped within the foam cells. This results in an elevation of internal pressure within the foam, ultimately leading to an enhancement in its mechanical properties.

## 4. Conclusions

In this study, EVA foam underwent static and dynamic compression tests. Through quasi-static and dynamic mechanical tests, it was observed that the stress of EVA increases with higher strain rates, indicating its sensitivity to strain rate. However, the strain rate sensitivity of EVA foam is less pronounced in the linear elastic stage, while it becomes more evident in the plateau stage and the densification stage. As the strain rate increases, the EVA foam absorbs more energy, but the energy absorption efficiency decreases. The energy absorption efficiency shows an increasing trend in the linear elastic stage and plateau stage, and starts to decrease when it enters the densification stage. The primary contributing factor to this behavior is the predominant influence of intracellular air during the plateau stage and the densification stage, while its impact during the linear elastic stage is minimal. To characterize the stress–strain response of EVA under the influence of intracellular air, a one-dimensional dynamic constitutive model incorporating an air leaking rate parameter was developed. There was a strong correlation observed between the fitted points and the experimental test results, indicating a high level of agreement. Furthermore, the dynamic buffering mechanism of EVA foam, influenced by the presence of air within the cells, can be effectively described through the utilization of this instanton equation. With increasing strain rates, the ratio of the air leaking rate to the strain rate gradually decreases, causing the air within EVA to be trapped in the cells rather than escaping in a timely manner. Consequently, this retention of air within EVA cells leads to an enhancement of its mechanical properties.

Developing an intrinsic model and analyzing its parameters to characterize the air action on the foam inside the cells is a viable approach. However, due to the intricate internal structure of the foam, experimental verification poses challenges. In the future, reliable simulation models can be constructed to facilitate accurate simulations and analyses.

## Figures and Tables

**Table 1:** Parameters of constitutive model in the above compression test.

Parameters	0.0017/s	0.0330/s	0.1700/s
$a_{1}$/MPa	0.0129 ± 1.4 × 10^−3^	0.0129 ± 1.4 × 10^−3^	0.0129 ± 1.4 × 10^−3^
$a_{2}$	20 ± 5.8 × 10^−2^	20 ± 5.8 × 10^−2^	20 ± 5.8 × 10^−2^
$\overset{\cdot }{\delta }$/s^−1^	3.28243 × 10^−4^ ± 7.4 × 10^−3^	3.72357 × 10^−3^ ± 1.2 × 10^−3^	0.001 ± 1.3 × 10^−3^
$\frac{\overset{\cdot }{\delta }}{\overset{\cdot }{\varepsilon }}$/s^−1^	0.19308	0.09758	0.00588
R^2^	0.999002	0.999514	0.998055

## Data Availability

Data is contained within the article.

## References

[1] Rinde J.A., Hoge K.G. (1971). Time and Temperature Dependence of the Mechanical Properties of Polystyrene Bead Foam. J. Appl. Polym. Sci..

[2] Nagy A., Ko W.L., Lindholm U.S. (1974). Mechanical Behavior of Foamed Materials Under Dynamic Compression. J. Cell. Plast..

[3] Liu D.-S., Chen Z.-H., Tsai C.-Y., Ye R.-J., Yu K.-T. (2017). Compressive Mechanical Property Analysis of Eva Foam: Its Buffering Effects at Different Impact Velocities. J. Mech..

[4] Li Y., Gong P., Liu Y., Niu Y., Park C.B., Li G. (2021). Environmentally Friendly and Zero-Formamide EVA/LDPE Microcellular Foams via Supercritical Carbon Dioxide Solid Foaming. ACS Appl. Polym. Mater..

[5] Chang B.P., Kashcheev A., Veksha A., Lisak G., Goei R., Leong K.F., Tok A.l.Y., Lipik V. (2023). Nanocomposite Foams with Balanced Mechanical Properties and Energy Return from EVA and CNT for the Midsole of Sports Footwear Application. Polymers.

[6] Cheng L., Liu S., Yu W. (2021). Recyclable Ethylene-Vinyl Acetate Copolymer Vitrimer Foams. Polymer.

[7] Gibson L.J., Ashby M.F. (1997). The Mechanics of Foams: Refinements. Cellular Solids: Structure and Properties.

[8] Shen J., Lu G., Ruan D. (2010). Compressive Behaviour of Closed-Cell Aluminium Foams at High Strain Rates. Compos. Part B Eng..

[9] Dannemann K.A., Lankford J. (2000). High Strain Rate Compression of Closed-Cell Aluminium Foams. Mater. Sci. Eng. A.

[10] Srivastava V., Srivastava R. (2014). On the Polymeric Foams: Modeling and Properties. J. Mater. Sci..

[11] Sadot O., Ram O., Anteby I., Gruntman S., Ben-Dor G. (2016). The Trapped Gas Effect on the Dynamic Compressive Strength of Light Aluminum Foams. Mater. Sci. Eng. A.

[12] Sherwood J., Frost C. (1992). Constitutive Modeling and Simulation of Energy Absorbing Polyurethane Foam Under Impact Loading. Polym. Eng. Sci..

[13] Liu Q.L., Subhash G. (2004). A Phenomenological Constitutive Model for Foams under Large Deformations. Polym. Eng. Sci..

[14] Walter T.R., Richards A.W., Subhash G. (2009). A Unified Phenomenological Model for Tensile and Compressive Response of Polymeric Foams. J. Eng. Mater. Technol.-Trans. ASME.

[15] Zhang J., Lin Z., Wong A., Kikuchi N., Li V.C., Yee A.F., Nusholtz G.S. (1997). Constitutive Modeling and Material Characterization of Polymeric Foams. J. Eng. Mater. Technol.-Trans. ASME.

[16] Yang L.M., Shim V.P.W. (2004). A Visco-Hyperelastic Constitutive Description of Elastomeric Foam. Int. J. Impact Eng..

[17] Anani Y., Alizadeh Y. (2011). Visco-Hyperelastic Constitutive Law for Modeling of Foam’s Behavior. Mater. Des..

[18] Jeong K.Y. (2016). Constitutive Modeling of Polymeric Foams Having a Four-Parameter Modulus Function with Strain Rate Sensitivity. J. Mech. Sci. Technol..

[19] Su B., Zhou Z., Xiao G., Wang Z., Shu X., Li Z. (2017). A Pressure-Dependent Phenomenological Constitutive Model for Transversely Isotropic Foams. Int. J. Mech. Sci..

[20] Li X., Tao J., Landauer A.K., Franck C., Henann D.L. (2022). Large-Deformation Constitutive Modeling of Viscoelastic Foams: Application to a Closed-Cell Foam Material. J. Mech. Phys. Solids.

[21] Wen Y., Lai Z., Ma J., Liu H., Wang Y., Chi H., Huang R. (2023). A Dynamic Constitutive Model for High-Density Rigid Polyurethane Foam Subjected to Impact Loading. Constr. Build. Mater..

[22] Xu S., Beynon J.H., Ruan D., Yu T.X. (2012). Strength Enhancement of Aluminium Honeycombs Caused by Entrapped Air under Dynamic Out-of-Plane Compression. Int. J. Impact Eng..

[23] Li Q.M., Magkiriadis I., Harrigan J.J. (2006). Compressive Strain at the Onset of Densification of Cellular Solids. J. Cell. Plast..

[24] Avalle M., Belingardi G., Montanini R. (2001). Characterization of Polymeric Structural Foams under Compressive Impact Loading by Means of Energy-Absorption Diagram. Int. J. Impact Eng..

[25] Lu F., Ren M., Gao D., Xi D. (2022). A new method for analyzing dynamic behavior of hyper-elastoplastic foam under continuous impact. J. Vib. Shock.

[26] Lu F., Hua G., Wang L., Jiang H., Gao D. (2019). A Phenomenological Constitutive Modelling of Polyethylene Foam under Multiple Impact Conditions. Packag. Technol. Sci..

[27] Zhang X.W., Yu T.X. (2009). Energy Absorption of Pressurized Thin-Walled Circular Tubes under Axial Crushing. Int. J. Mech. Sci..

